# Pulmonary embolism originating from germ cell tumor causes severe left ventricular dysfunction in a healthy young adult with full recovery: a case report

**DOI:** 10.1186/s12872-021-02066-7

**Published:** 2021-05-26

**Authors:** Khaled Elenizi, Rasha Alharthi, Michel Galinier

**Affiliations:** 1 Department of Cardiology, CHU-Toulouse, University Hospital of Toulouse, Hôpital Rangueil, 1 Avenue Jean Poulhès, 31059 Toulouse, France; 2 Department of Internal Medicine, College of Medicine, Prince Sattam Bin Abdulaziz University, Alkharj, 11942 Saudi Arabia

**Keywords:** Pulmonary embolism, Heart failure, Testicular cancer, Left ventricle, Case report

## Abstract

**Background:**

Cancer associated thrombosis is recognized. However, pulmonary embolism (PE) from testicular cancer is rarely reported. Right ventricular (RV) function and PE are closely related. The RV cannot cope with a sudden increase in afterload because of PE and this causes dysfunction, but isolated left ventricular dysfunction in this context is not reported in the literature.

**Case presentation:**

We report an unusual association of pulmonary embolism and testicular germ cell tumor complicating severe left heart failure and full recovery at three months follow up in a 33-year-old patient with no prior medical history. The diagnosis was made after comprehensive history taking and physical examination with the help of different imaging modalities. Full recovery was achieved after optimal medical therapy.

**Conclusion:**

This case raises our awareness of unusual clinical presentation as we report associated left-sided severe heart failure in cancer-related pulmonary embolism. Pulmonary embolism in healthy young adults warrant in-depth causative exploration.

## Background

Pulmonary embolism (PE) affects around 430,000 individuals in Europe [1] and between 300,000 and 600,000 individuals in the United states each year [2]. Night percent of cases are caused by deep vein thrombosis (DVT) [3]. Cancer increases PE risk by 4.1 folds [4]. When DVT migrate to the pulmonary circulation, it’s called venous thromboembolism (VTE). Predisposing risk factors such as recent trauma, surgery, hospitalization or immobility can be identified in 50% of cases [5]. In case of unprovoked PE, search for thrombophilia state or cancer is warranted [6, 7]. Thrombosis and cancer have a strong relationship with different mechanisms in thromboembolic events [8]. Cancer patients represent 20% of pulmonary embolism cases and in 15% of all cancer patients VTE is present [9]. Tissue factor-factor VII complex is known to be involved in the mechanism of hypercoagulability in cancer patient [10] while factor II is not. The most dangerous and life-threatening clinical manifestation of VTE is PE and could lead to cardiogenic shock. Direct relation to RV dysfunction and PE is well established, but no direct association in literature is found for LV dysfunction. We report an unusual clinical scenario for left-sided heart failure diagnosed in the setting of PE for which testicular cancer was the only relevant predisposing factor in a healthy young adult.

## Case presentation

A 33-year-old male smoker with no prior medical history came to the emergency department with sudden onset dyspnea and hemoptysis associated with lower limb edema. The physical examination found: heart rate 125 bpm, regular sinus rhythm with no added sounds, blood pressure 240/130 mmHg, polypnea at 45 breaths per minute, normal oxygen saturation, limited bilateral air entry with crepitation, lower limbs pitting edema, hepatojugular reflex, no evidence of deep vein thrombosis, normal abdominal examination. Biological markers were hemoglobin 15 g/dl, white blood cells 16 G/l, CRP 87 mg/L, ProBNP 3612 pg/mL, stable troponin at 76 ng/ml, sodium 133 mmol/L, potassium 4.7 mmol/L, creatinine 100 μmol/L.

## Investigations

**Fig. 1:**
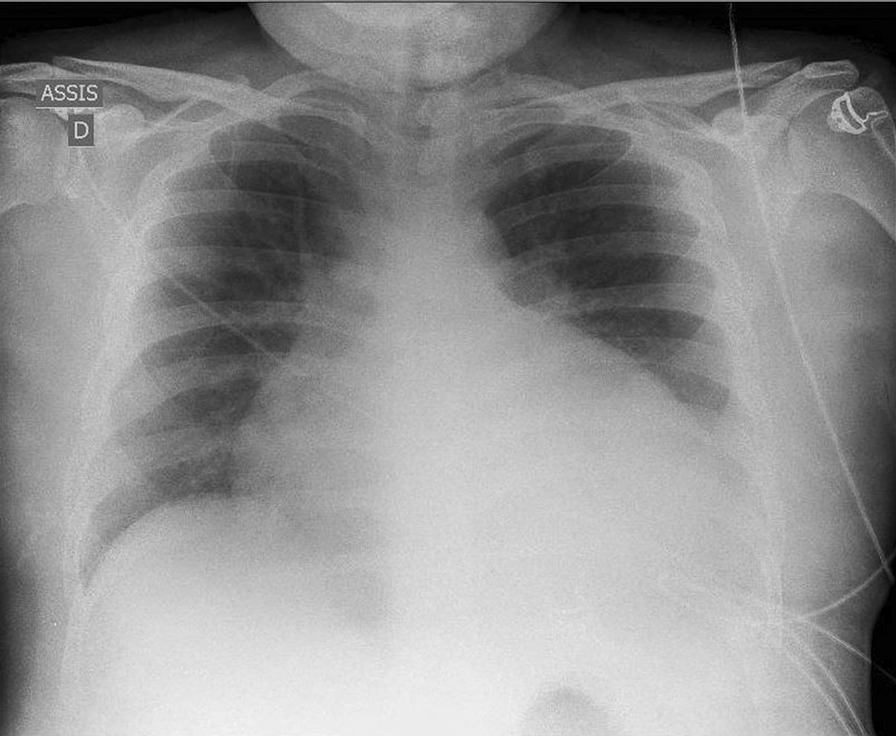
Chest x-ray shows cardiomegaly with bilateral hilar congestion

**Fig. 2:**
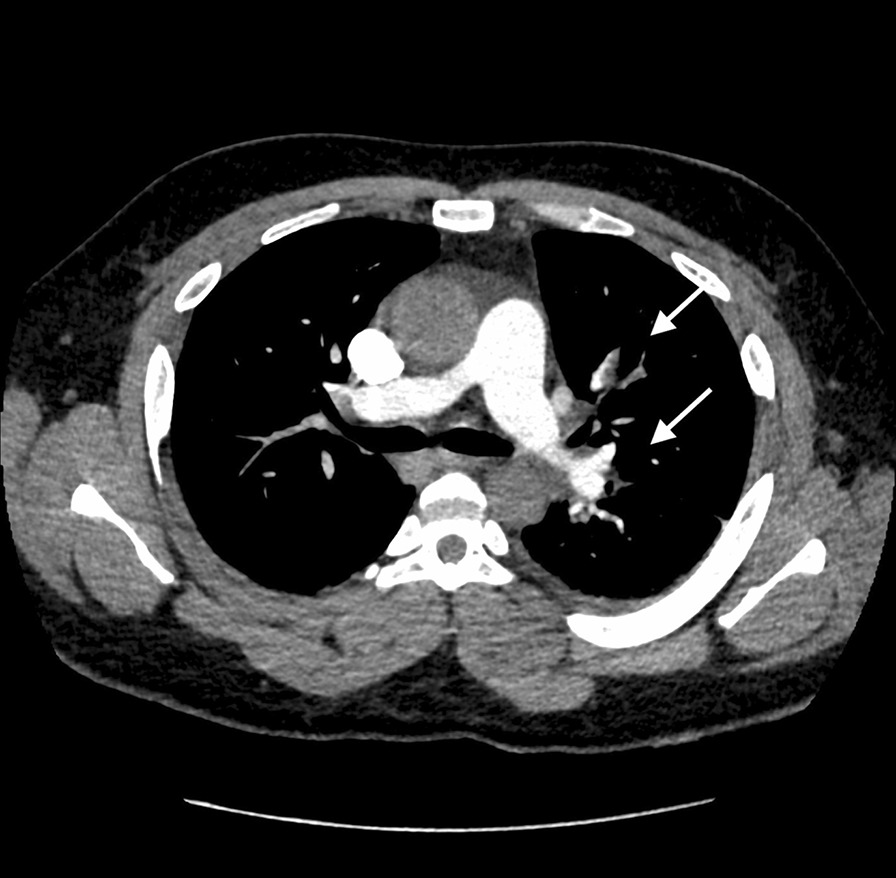
Computed tomography (CT) pulmonary angiogram shows distal pulmonary embolisms in inferior lingular segment at the upper lobe, lateral and posterior segments in lower lobe

**Fig. 3:**
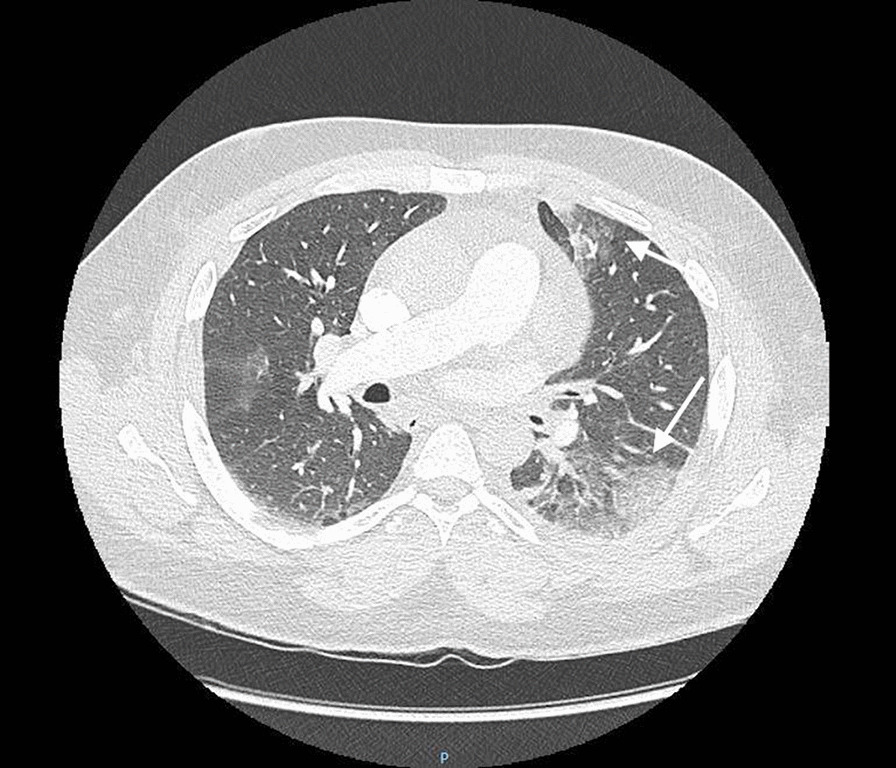
Computed tomography (CT) of the pulmonary parenchyma shows associated infarctions corresponding with ischemic lesions

**Fig. 4:**
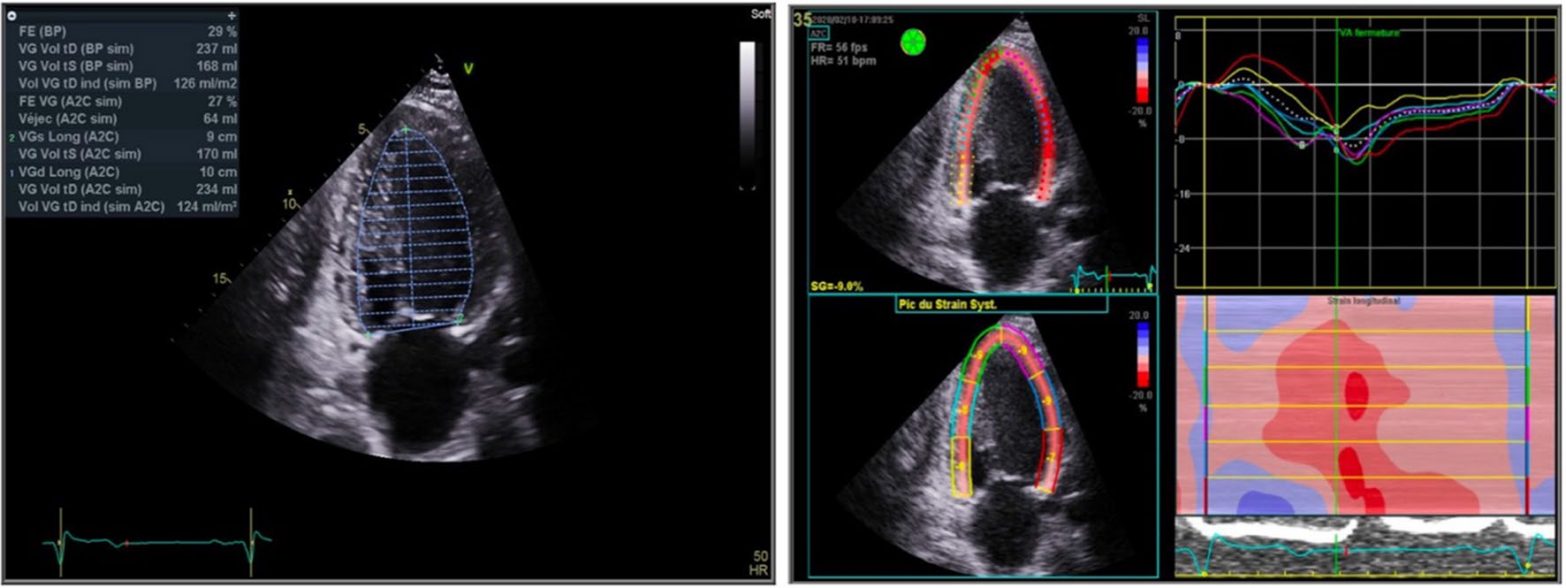
Transthoracic echocardiography shows LV parameters (left) and LV strain (right)

**Fig. 5:**
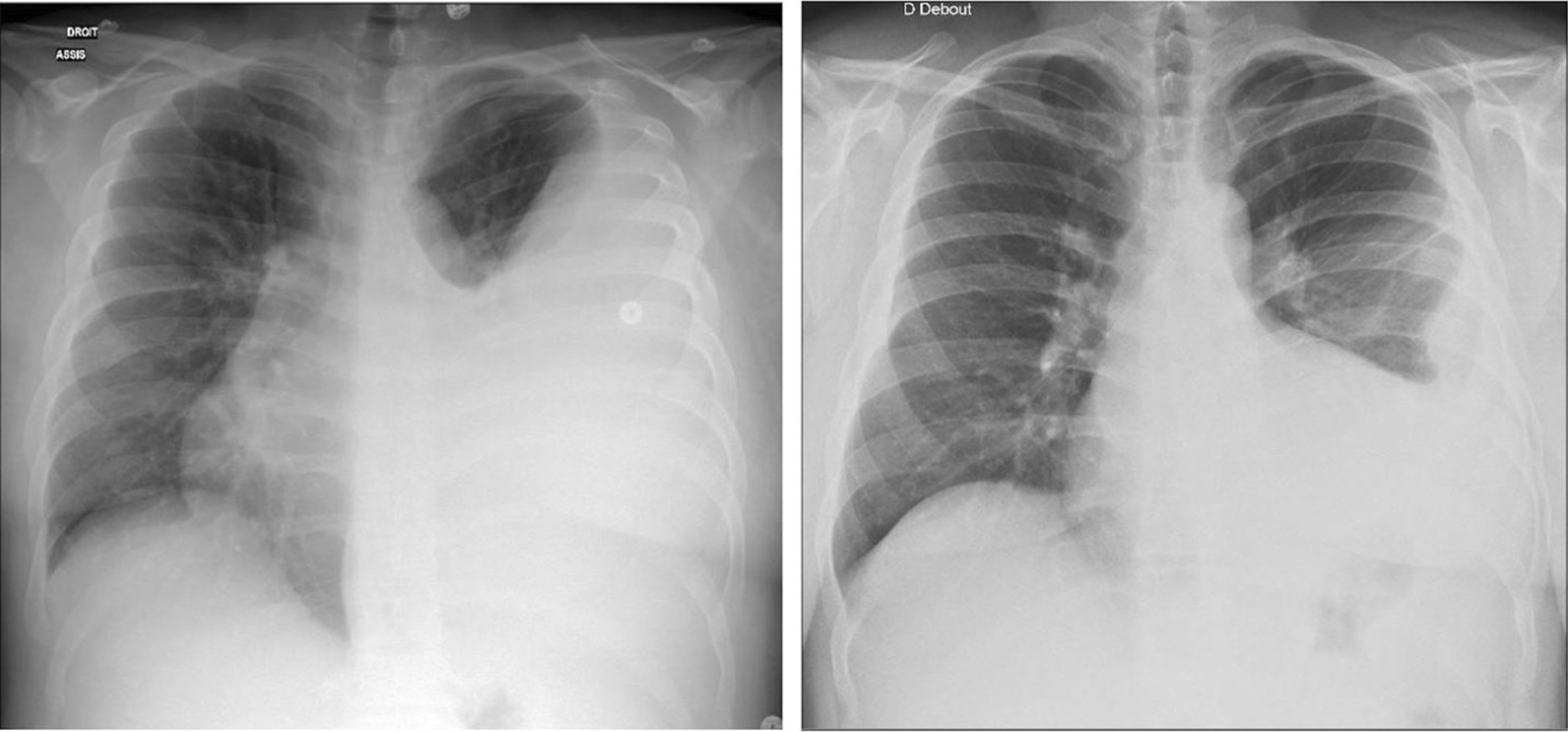
Chest x-ray shows left pleural effusion with superinfection before drainage (left) after drainage (right)

ECG showed regular sinus tachycardia at 110 bpm, ST depression and T waves inversion in inferno-lateral leads. Chest x-ray showed cardiomegaly with bilateral hilar congestion (Fig. 1). Computed tomography (CT) pulmonary angiogram revealed distal pulmonary embolisms in inferior lingular segment at the upper lobe, lateral and posterior segments in lower lobe with associated infarctions (Figs. 2 and 3). Transthoracic echocardiography revealed dilated cardiomyopathy, left ventricular indexed end diastolic volume 237 ml/m^2^, end systolic volume 168 ml, global hypokinesia, LVEF 29%, LV strain -9%, moderate functional mitral regurgitation, right ventricle with normal function, TAPSE 20 mm, PAPS 45 mmHg, low cardiac output 3.4 l/ min (Fig. 4). Patient subsequently underwent Coronarography that revealed no significant coronary artery disease and LVEF at 27% on ventriculography. The assessment of LV global hypokinesia was completed with cardiac MR that showed dilated cardiomyopathy indexed end diastolic volume 200 ml/m^2^, no late gadolinium enhancement, no evidence of other anomalies. Patient subsequently developed left pleural effusion with superinfection drained 2 times of inflammatory serohematic fluid but no neoplastic cells were found (Fig. 5).

## Differential diagnosis

**Fig. 6:**
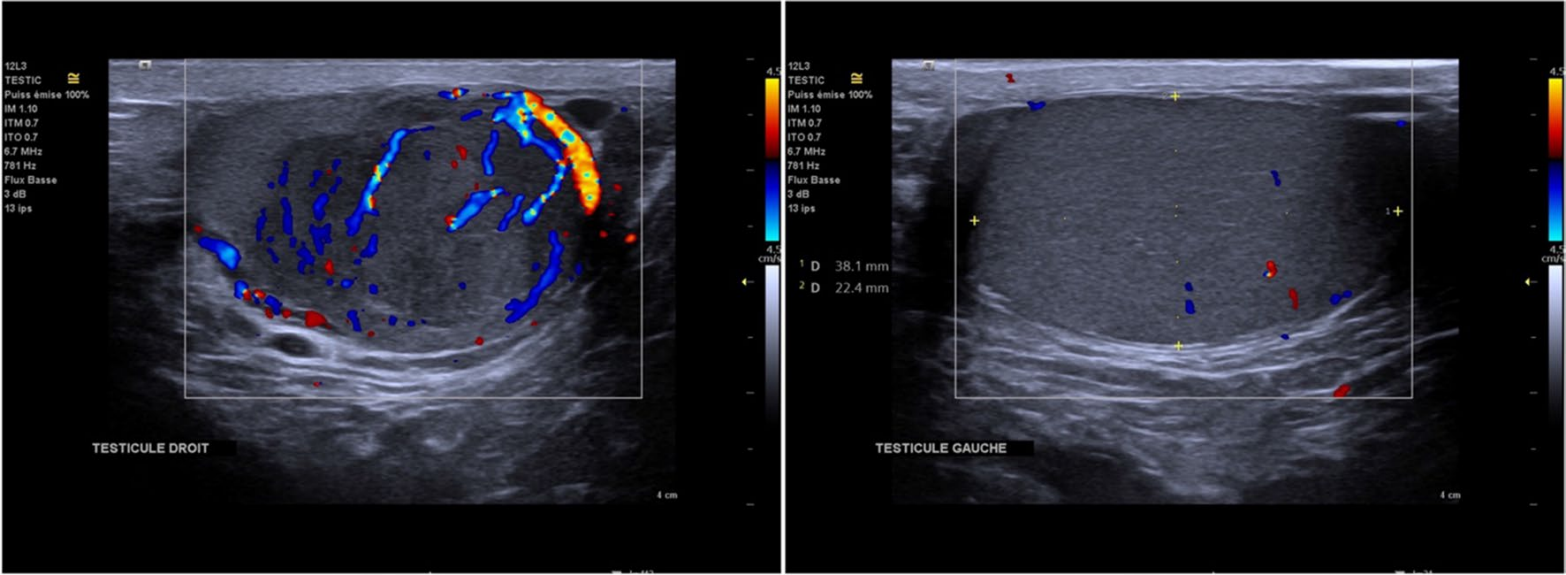
Testicular ultrasound shows right testicular vascularized mass with calcification (left) normal left testicle (right)

To look for the cause of pulmonary embolism an extensive workup was carried out with computed tomography (CT) of the abdomen, pelvis and brain showing no abnormalities. Testicular ultrasound showed vascularized right testicular mass with calcifications measuring 28X23 mm (Fig. 6). Genetic testing found Testicular germ cell tumor classification UICC/TNM 2016 pT1, R0. Beta Human chorionic gonadotrophin (β HCG) measured at 39 UI/L, alpha fetoprotein (AFP) at 3 ng/ml and lactate dehydrogenase (LDH) 183 U/L all normal. Hematological testing found Factor II Mutation with heterozygous Factor II genotype; Factor V Leiden was normal. No etiological origin was found for the LV dysfunction except PE.

## Treatment

Heart failure with reduced ejection fraction treatment was started with ramipril 2.5 mg once daily, spironolactone 25 mg once daily, bisoprolol 5 mg once daily and diuretics. Intermediate high-risk pulmonary embolism treatment was shifted from heparin to Xarelto after assuring haemodynamic stability. Testicular cancer was treated with right orchidectomy through an incision in the lower lateral abdomen.

## Outcome and follow-up

At 3 months follow up, the patient showed great improvement with the following parameters; LVEF from 29 to 55%, LV telediastolic volume normalized from 237 to 100 ml/m^2^, LV strain from − 9 to − 15% with the disapearance of the pulmonary hypertension. In this context, no therapeutic modification was made with no indication for ICD. The patient stated a big improvement in symptoms in this period.

## Discussion and conclusion

Testicular cancer accounts for around 1–1.5% of male cancers and affects younger males in their third or fourth decade of life mainly [11]. Testicular cancer is not on the top list of thrombogenic cancers but is a possible source of neoplasm-related coagulopathy [12].Cancer therapy is a major cause of VTE but our patient was not diagnosed before [13]. VTE in cancer has a complex pathophysiology but result generally from coagulation factors activation [14]. Inheriting one copy of the mutation in the F2 gene raises the chance of 2 or 3 in 1000. Persons who inherit two copies of the gene, one from each parent, may be at risk as much as 20 in 1000. [15]. Thrombophilia due to factor 2 gene mutation is considered hereditary with no cancer association and manifest mainly by DVTs [16]. In our case, we found heteozygote FII mutation rising the quetion if it is an accidental or a causative realtion.

To our awareness, only one case has been reported where PE was the first manifestation of testicular cancer [17], but surprisingly our case has left-sided severe heart failure and PE as the first manifestation of testicular cancer. Comprehensive history taking remains the ultimate tool in the search for unusual underlying pathologies, as testicular cancer has been identified in our case due to comprehensive interrogation.

Suitable diagnostic imaging modality is of great importance, as full body scan in our patient has not been able to detect the testicular mass and ultrasonography was the right diagnostic tool.

In our case, optimal medical therapy resulted in complete recuperation of left ventricular dysfunction and disappearance of the pulmonary hypertension within 3 months. We do not know whether PE has a cause - effect relationship with LV dysfunction or it was simply a triggering event in a previously failing heart.

## Learning points/take home messages

1. Cancer should be considered as a cause of pulmonary embolism even at young ages
2. Comprehensive history taking is very important
3. Multimodality imaging is helpful for diagnosis and management
4. Awareness of unusual clinical associations

## Data Availability

All data are available in the manuscript.

## Abbreviations

PE: Pulmonary embolism
RV: Right ventricle
LV: Left ventricle
DVT: Deep venous thrombosis
VTE: Venous thromboembolism
CT: Computed tomography
LVEF: Left ventricular ejection fraction
TAPSE: Tricuspid annular plane systolic excursion
PAPS: Systolic pulmonary arterial pressure
MR: Magnetic resonance
OD: Once daily
ICD: Intracardiac defibrillator
PHT: Pulmonary hypertension
